# Transcriptomics and Metabolomics Analyses Provide Novel Insights into Glucose-Induced Trophic Transition of the Marine Diatom *Nitzschia laevis*

**DOI:** 10.3390/md19080426

**Published:** 2021-07-27

**Authors:** Xuemei Mao, Mengdie Ge, Xia Wang, Jianfeng Yu, Xiaojie Li, Bin Liu, Feng Chen

**Affiliations:** 1 Shenzhen Key Laboratory of Marine Microbiome Engineering, Institute for Advanced Study, Shenzhen University, Shenzhen 518060, China; maoxm@szu.edu.cn (X.M.); gemengdie2020@email.szu.edu.cn (M.G.); xiawang6632@szu.edu.cn (X.W.); j.yu@szu.edu.cn (J.Y.); lixiaojie@szu.edu.cn (X.L.); 2 College of Physics and Optoelectronic Engineering, Shenzhen University, Shenzhen 518060, China; 3 Institute for Innovative Development of Food Industry, Shenzhen University, Shenzhen 518060, China

**Keywords:** diatom, fucoxanthin, eicosapentaenoic acid (EPA), transcriptomics, trophic transition

## Abstract

Diatoms have important ecological roles and are natural sources of bioactive compounds. *Nitzschia laevis* is a member of marine diatoms that accumulates high-value products including fucoxanthin and eicosapentaenoic acid (EPA). In this study, physiological data showed that comparing to autotrophic growth, mixotrophic cultivation with glucose supplementation led to a decrease of chlorophyll and fucoxanthin content in *N. laevis*, and an increase of biomass density and EPA yield. To further examine the metabolic barriers for fucoxanthin and EPA biosynthesis, comparative transcriptomic and metabolome analyses were conducted, with a focus on the genes related to carotenoids biosynthesis and fatty acid metabolism. The results indicated that phytoene desaturase (PDS) and zeta-carotene isomerase (ZISO) could be the rate-limiting enzymes in carotenoid biosynthesis. The transcription regulation of 3-ketoacyl-CoA synthase (KCS) and elongation of very long chain fatty acids protein (EVOVL) are important contributors associated with polyunsaturated fatty acids (PUFAs) accumulation. Furthermore, we also investigated the glucose-associated regulatory genes using weighted gene co-expression network analysis, and identified potential hub genes linked with cell cycle, carbohydrate metabolism, purine biosynthesis, and lipid metabolism. This study offers a high-quality transcriptome resource for *N. laevis* and provides a molecular framework for further metabolic engineering studies on fucoxanthin and EPA production.

## 1. Introduction

Diatoms are important producers in the aquatic systems, as they are estimated to undertake 20–25% of the global carbon fixation and contribute ~40% of net primary productions [1,2]. They also play important roles in taking part in the biogeochemical cycling of silicon and carbon in the ecosystem [3]. Diatoms could grow under phototrophic conditions using CO_2_ as the carbon source and light as energy. Some diatoms have developed strategies in the form of mixotrophic or heterotrophic growth to utilize organic carbon sources such as glucose, glycerol in certain niches. Diatoms are important sources of bioactive compounds like fucoxanthin, polyunsaturated fatty acids (PUFAs), e.g., eicosapentaenoic acid (EPA), flavonoids, phenolic compounds, as well as lipid production for biofuels [4,5]. Fucoxanthin, one of the most abundant carotenoids in diatoms, has promising applications in human health due to its antioxidant, anti-inflammatory, anticancer, anti-obesity, antidiabetic, antiangiogenic, and antimalarial activities [6,7]. EPA, a bioactive PUFA, plays a cardioprotective role in preventing the occurrence of cardiovascular diseases [8]. Chrysolaminarin (β-1,3-glucan), the principal storage polysaccharide in the diatom stored in vacuoles, has anti-tumor bioactivity [9]. Chrysolaminarin and lipids are the two major storage substances for carbon skeletons in diatoms; lipids (primarily triacylglycerols (TAGs)) are also considered as potential sources for biofuels [10].

The morphology and physiology of diatoms are distinctive to other photosynthetic organisms, especially on their intracellular compartmentation and carbon partitioning pathways due to its complex evolutionary history [11]. Several diatom species have been used as research models, including a centric diatom *Thalassiosira pseudonana* for silica biomineralization, and a pennate diatom *Phaeodactylum tricornutum* for carbohydrate metabolism, xanthophyll cycle, and lipid metabolism [12,13]. With the advances in genomic sequencing and multi-omics analyses, the metabolic pathways in the model diatoms are being elucidated, and genetic engineering approaches have been developed [13,14,15,16]. One of the challenges is that the metabolism of diatoms is complex and substantially different among diatom species [11], therefore some questions could not be demonstrated in existing model systems. For example, *P. tricornutum* is unable to assimilate glucose due to the lack of a transporter gene [17]. *Nitzschia laevis* can grow under autotrophic, mixotrophic, and heterotrophic conditions, and reach high cell density when glucose is supplemented as the sole carbon resource [18,19]. Thus, *N. laevis* is likely to have unique metabolic characteristics during the glucose-induced trophic transition. In addition, *N. laevis* has also been demonstrated to be a robust industrial strain for fucoxanthin and EPA production [20], thus it is an important organism to be investigated and further optimized for industrial.

Glucose is an important source of carbon to many organisms, it has been reported that the assimilation of glucose has a profound impact to the gene expression pattern, metabolic networks and cell growth in higher plants and green algae [21,22]. The physiological impact of glucose assimilation to diatoms is yet clear. Previously, we found that supplementation of glucose could induce a shift of physiological parameters, e.g., growth rate and biomolecular composition; to elucidate the underlying molecular mechanisms that associated with growth trophic transitions in response to glucose, we conducted metabolomics and transcriptomics analyses, the data are presented in this work.

## 2. Results and Discussion

### 2.1. Physiological Changes in N. laevis under Glucose Addition

**Figure 1:**
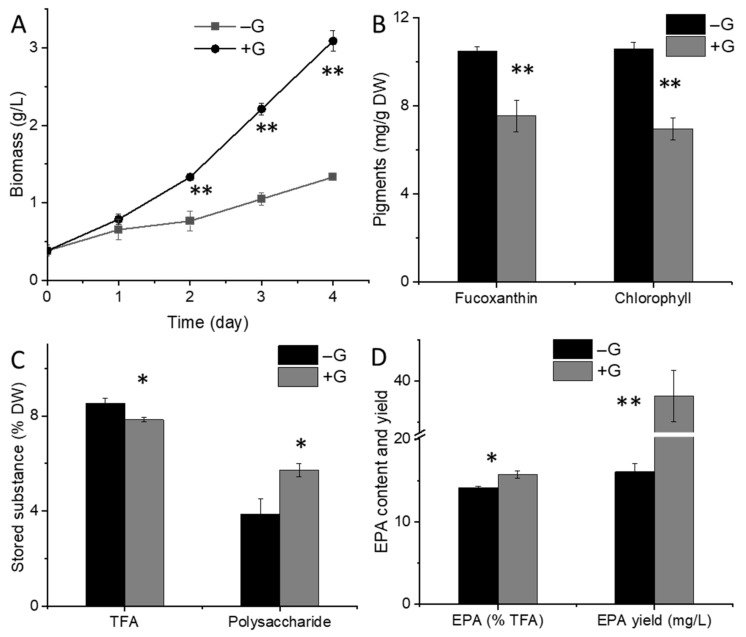
Physiological changes in *N. laevis* after glucose addition. (**A**) Biomass growth, (**B**) fuco-xanthin and total chlorophyll content, (**C**) total fatty acids (TFA) and intracellular soluble polysaccharides content, and (**D**) eicosapentaenoic acid (EPA) content and yield after 4-days culture. * indicates *p* < 0.05, and ** indicates *p* < 0.01. +G, with glucose addition; −G, without glucose addition.

With the addition of glucose (+G), the cultivation shifted from autotrophy to mixotrophy (or pure heterotrophy), resulting in a significant increase in biomass yield in comparison to the control group (−G) (Figure 1A) (*p* < 0.01). Moreover, the cellular abundance of fucoxanthin was decreased in mixotrophic cells after 4-day culture with glucose (Figure 1B). In diatom, fucoxanthin and chlorophyll a/c are primary pigments in the light-harvesting complex associated with photosystem I and II [23,24]. The decrease of both chlorophyll (Figure 1B) and fucoxanthin content indicates a general downregulation of the photosynthetic apparatus (including PSI and PSII) under glucose addition. TFA content displayed a minor decrease on Day 4 (Figure 1C). As both lipid and carbohydrates production were dependent on the carbon precursors and used as stored substances, the synthesis of these biomolecules are potential competitors for carbon backbones. In microalgae, the most abundant carbohydrate molecules are species-dependent, as that in cyanobacteria is glycogen, whereas that in green algae Chlamydomonas is starch [25]. The main carbohydrate in diatom like *Phaeodactylum*, is chrysolaminaran [26]. In this study, the intracellular soluble polysaccharides, likely in the form of chrysolaminaran, were analyzed and showed a significant increase upon glucose addition (Figure 1C) (*p* < 0.05). The percentage of EPA in TFA was increased on Day 4 with glucose addition (Figure 1D), and the EPA yield reached 38.18 mg/L in +G group while only 16.09 mg/L in −G group (*p* < 0.01). A previous study also has concluded that mixotrophic culture is preferred for the production of EPA from *N. laevis* [27].

### 2.2. The Changes of Transcriptome during the Transition from Autotrophy to Mixotrophy

**Figure 2:**
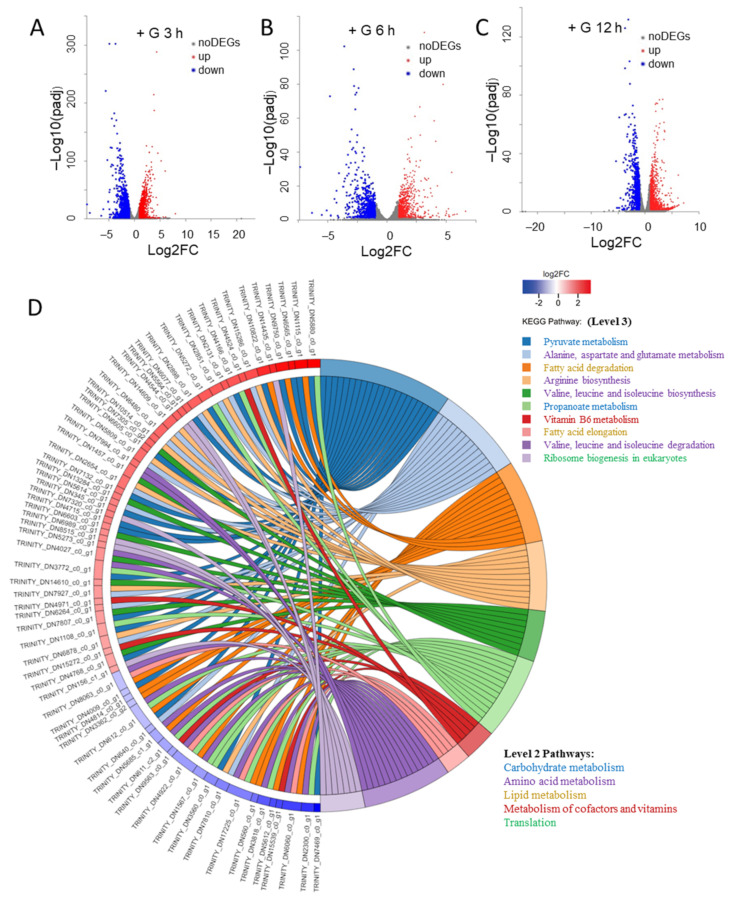
Differentially expressed genes (DEGs) overview during the transition from autotrophy to mixotrophy. (**A**) The volcano plots of DEGs at 3 h. (**B**) The volcano plots of DEGs at 6 h. (**C**) The volcano plots of DEGs at 12 h. (**D**) KEGG pathways enrichment for the DEGs in the +G3h group. DEGs, differential expressed genes; FC, fold change.

RNA-seq is a powerful tool to provide insights to gene expression and function, the association of genes to metabolic pathways, and their regulations. Data generated from RNA-seq can be used to complement genomic studies for functional annotations and evolution of gene clusters and metabolic pathways. To elucidate the underlying mechanisms that associated with physiological changes in *N. laevis*, we investigated transcriptome-wide differences in gene expression during the transition from autotrophy to mixotrophy. Samples were collected for RNA-seq at 3 h, 6 h, and 12 h from both the glucose group (with glucose addition, +G) and control group (without glucose addition, +G). De novo sequences were prepared from 18 samples (referred as +G3h, +G6h, +G12h, −G3h, −G6h, −G12h). The de novo assembly generated 16,622 unigenes in total (Table S1). To reflect the gene expressional correlation between samples, the Pearson correlation coefficients for all gene expression levels between two samples were shown in Table S2. Moreover, the principal component analysis (PCA) results also revealed the relationship between the samples (Figure S1A). The transcription profiles among +G and −G groups showed significant divergence, and time points also attributed to the divergence among groups, while the three biological replicates were comparable. The box plot showed an overview of the gene expression level in each sample (Figure S1B). Through the sequence similarity search in NR (Non-Redundant Protein Sequence) database, functions information of genes was predicted and the similarity between the transcript sequences of *N. laevis* and the similar species was obtained. As shown in Figure S1C, 3163 genes were matched with homologous genes from *Pseudo-nitzschia multistriata*, and 2665 homologous genes were identified in *Fragilariopsis cylindrus*. Moreover, these genes accounted for ~52.8% of total genes, and genes conserved across all three species of *Phaeodactylum tricornutum*, *Fistulifera solaris*, and *Thalassiosira oceanica* accounted for ~37.0% of total genes. These results revealed the close phylogenetic links between *N. laevis* and other diatoms. The abundance of transcripts from +G group were compared with respective counterparts from –G group, and interpreted as log_2_FoldChange (FC), and genes with log_2_FC ≥ 1 or ≤ −1 and padj ≤ 0.05 were defined as differentially expressed genes (DEGs). The volcano plots shown the distribution of DEGs (Figure 2A–C): There were 1068 upregulated DEGs and 1058 downregulated DEGs in the +G3h group, 699 upregulated DEGs and 728 downregulated DEGs in the +G6h group, and 822 upregulated DEGs and 781 downregulated DEGs in the +G12h group. As the +G3h group yielded the largest number of DEGs, the KEGG pathways enrichment for the DEGs of +G3h group was performed and pathways significantly enriched (*p* < 0.05) were shown in Figure 2D. The top 10 enriched pathways of DEGs were listed with relative DEG IDs, including several amino acid metabolism pathways (‘alanine, aspartate and glutamate metabolism’, ‘arginine biosynthesis’, ‘valine, leucine and isoleucine biosynthesis’, ‘valine, leucine and isoleucine degradation’); two carbohydrate metabolism pathways (‘pyruvate metabolism’, ‘propanoate metabolism’); two lipid metabolism pathways (‘fatty acid degradation’, ‘fatty acid elongation’); one pathway of translation (‘ribosome biogenesis in eukaryotes’); and ‘vitamin B6 metabolism’.

To identify gene families that quickly respond to the transition from autotrophy to mixotrophy, the 100 most up- and downregulated DEGs at 3 h were listed for hierarchical cluster analysis to group these DEGs with their expressional patterns during the first 12 h (Figure S2A,B). Even though most of these DEGs were unknown in function according to sequence alignment results in NR and KEGG database, some genes were annotated and classified. The results showed that most of these DEGs were associated with carbohydrate metabolism, amino acid metabolism, and lipid metabolism (Figure S2C,D). For carbohydrate metabolism, most of upregulated DEGs were involved in pyruvate metabolism and glycolysis/gluconeogenesis, while most of downregulated DEGs were involved in fructose and mannose metabolism, glyoxylate and dicarboxylate metabolism, and propanoate metabolism (Table S3). For lipid metabolism, one gene in fatty acid biosynthesis, one in glycerophospholipid metabolism and one in glycerolipid metabolism were downregulated, while three genes in fatty acid degradation, two genes in biosynthesis of unsaturated fatty acids, and one in fatty acid biosynthesis were upregulated (Table S3). Besides, note that there were 2 genes (hexaprenyl-diphosphate synthase (hexPS) and diphosphomevalonate decarboxylase (MVD)) related in terpenoid backbone biosynthesis pathway in the list of top 100 upregulated DEGs (Table S3), and terpenoid backbone biosynthesis is directly associated with carotenoids biosynthesis, thus influence the production of fucoxanthin.

### 2.3. The Changes of Metabolites Profile during the Transition from Autotrophy to Mixotrophy

**Figure 3:**
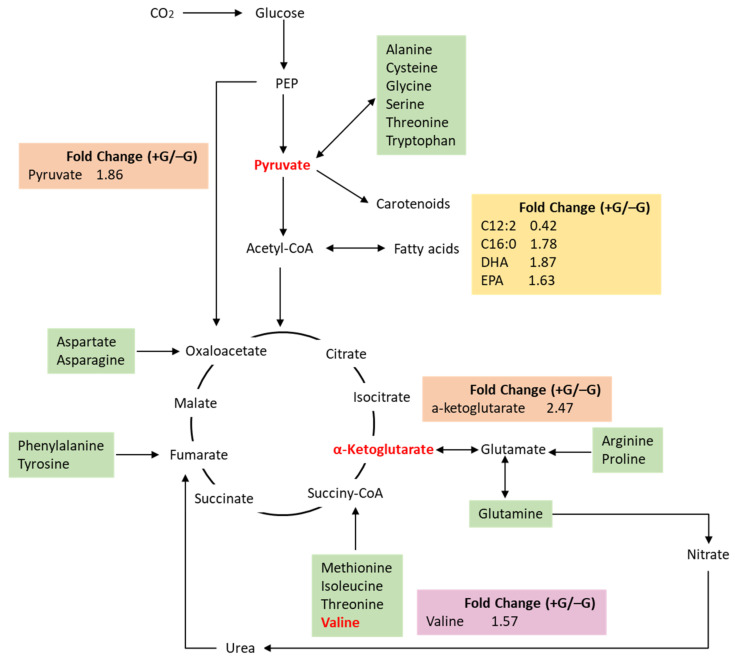
Brief overview of amino acid and central carbon metabolism based on metabolomic analysis. The red color of words indicates the content of the metabolite was increased under glucose addition.

Metabolic pathways in microalgae are responsive to environmental changes. Even with transcriptomic data, biochemical regulation is subject to many other regulatory controls (e.g., regulated degradation, post translational modification, and allostery), so the conclusions remain tentative and hypothesis-generating rather than conclusive. However, the metabolomic data greatly strengthen the hypotheses produced by the transcriptomic data. Mass spectrometry were used to collect data on metabolites analysis, by applying a filtering coefficient of 30% variation of peak size, there were 13,139 and 1316 peaks identified under Positive ion mode (ESI+) and negative ion mode (ESI−), respectively (Table S4). To illustrate the differences of metabolomics between −G and +G groups, metabolites with fold change ≥1.2 or ≤0.83, and *p*-value <0.05 were defined as having significant changes. In total, there were 847 decreased metabolites and 856 increased metabolites under ESI+ in the +G microalgal cells (Figure S3A); while the signals of 136 metabolites under ESI- were decreased and that of 122 metabolites were increased (Figure S3B). With the exception of unknown compounds, most of these differential metabolites belong to the following chemical groups: lipids and lipid-like molecules, benzenoids, organoheterocyclic compounds, and organic acids (Table S4). As designated in the KEGG databases, most of identified metabolites belong to amino acid metabolism, metabolism of cofactors and vitamins, carbohydrate metabolism and lipid metabolism pathway (Figure S3C). In contrast to that of −G group, the valine content was increased by 57% (Figure 3). Valine is present in many proteins, mostly in the interior of globular proteins helping to determine the three-dimensional structure [28]. α-Ketoglutarate was significantly increased by 147% in the +G group, which is an intermediate compound of the tricarboxylic acid cycle (TCA cycle) and amino acid metabolism, nitrogen metabolism. Correlated with glucose addition, pyruvate, the product of glycolysis and precursor of carotenoids, fatty acids and many amino acids, was increased by 86% (Figure 3). Due to the crosstalk between amino acids and central carbon metabolism, the increase of valine, α-ketoglutarate, and pyruvate was consistent with the upregulation of several amino acid metabolic pathways revealed by transcriptomic analysis, including ‘valine, leucine and isoleucine biosynthesis’, ‘arginine biosynthesis’ and ‘alanine, aspartate and glutamate metabolism’. The upregulation of amino acid biosynthesis under mixotrophic culture was expected, as after glucose addition, amino acids and proteins synthesis were required for cell proliferation. The contents of C16:0, EPA, and DHA were greatly increased, while C12:2 was significantly decreased (Figure 3), indicating that the addition of glucose enhanced the biosynthesis of long-chain fatty acids. This result was also consistent with the transcriptomic result that fatty acid elongation pathway was upregulated by glucose addition.

### 2.4. Identification of the Candidate Genes Involved in Carotenoids Biosynthesis and Their Expression Profiles

From this section, we focused on the transcriptome results of selected functional pathways involved in fucoxanthin and fatty acid biosynthesis. Carotenoids biosynthesis includes two metabolic steps: terpenoid backbone biosynthesis and carotenoid biosynthesis. Carotenoids are derived from terpene backbone precursors, isopentenyl pyrophosphate (IPP) and dimethylallyl pyrophosphate (DMAPP). Previous studies have revealed that green microalgae produce IPP and DMAPP via the chloroplastic 2-C-methylerythritol 4-phosphate (MEP) pathway [29,30], while diatoms and higher plants commonly have both cytosolic mevalonate (MVA) pathway and chloroplastic MEP pathway for IPP and DMAPP synthesis [31,32]. These two pathways are likely to generate precursors that are allocated to different isoprenoid end-products. It is currently known that mono- and diterpenes are produced in the plastid compartment via the MEP pathway; whereas the cytosolic MVA pathway is used for the biosynthesis of triterpenes, including the sterol precursor, cycloartenol; however, the exchange of terpene precursors between the two compartments increased the complexity of studying their metabolism [33]. In this study, all genes encoding the enzymes of the MEP pathway and most genes of the MVA pathway were identified in *N. laevis* (Table S5). This study is the first report on the genes and expression profiles of the MEP and the MVA pathways in *N. laevis*.

**Figure 4:**
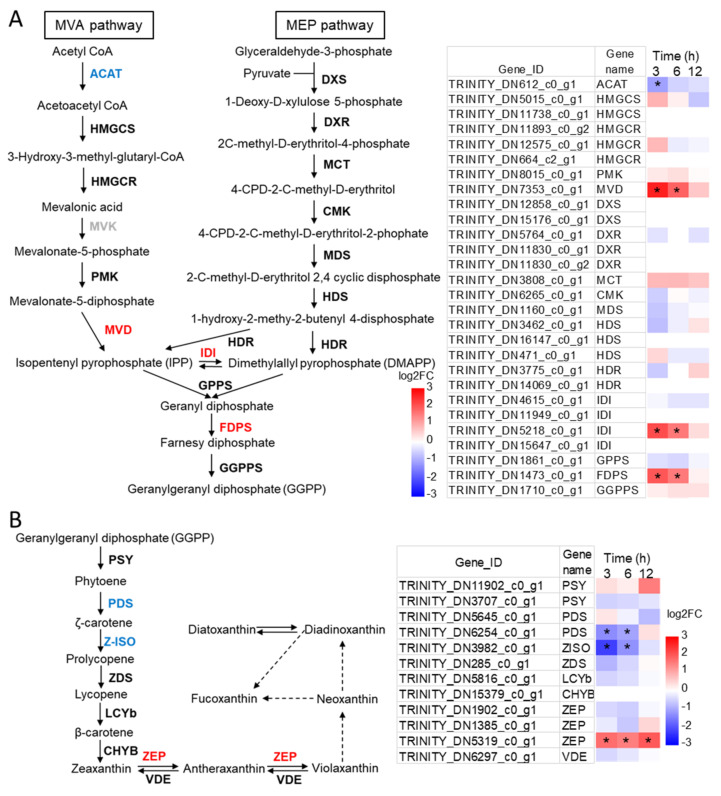
Expression patterns of genes in the carotenoid biosynthesis pathway. (**A**) Terpenoid backbone biosynthesis, (**B**) Carotenoid biosynthesis. ACAT, acetyl-CoA C-acetyltransferase; HMGCS, hydroxymethylglutaryl-CoA synthase; HMGCR, hydroxymethylglutaryl-CoA reductase; PMK, phosphomevalonate kinase; MVD, diphosphomevalonate decarboxylase; DXS, 1-deoxy-D-xylulose-5-phosphate synthase; DXR, 1-deoxy-D-xylulose-5-phosphate reductoisomerase; MCT, 2-C-methyl-D-erythritol 4-phosphate cytidylyltransferase; CMK, 4-diphosphocytidyl-2-C-methyl-D-erythritol kinase; MDS, 2-C-methyl-D-erythritol 2,4-cyclodiphosphate synthase; HDS, (E)-4-hydroxy-3-methylbut-2-enyl-diphosphate synthase; HDR, 4-hydroxy-3-methylbut-2-enoyl diphosphate reductase; IDI, isopentenyl-diphosphate Delta-isomerase; FDPS, farnesyl diphosphate synthase; GGPPS, geranylgeranyl diphosphate synthase; GPPS, geranyl diphosphate synthase; ZDS, zeta-carotene desaturase; PSY, phytoene synthase; PDS, phytoene desaturase; LCYb, lycopene beta-cyclase; ZEP, zeaxanthin epoxidase; VDE, violaxanthin de-epoxidase; ZISO, zeta-carotene isomerase; CHYB, beta-carotene 3-hydroxylase. * indicates the expression level in +G group was significantly differential compared with control group (|log_2_FC (Fold change)| ≥ 1.00 and padj < 0.05). The red color of genes indicates upregulation and blue color indicates downregulation.

The transcription level of genes relate to terpene precursors and carotenoid biosynthesis were further illustrated with the respective time points (Figure 4). All genes in MEP pathway showed no significant change through the 12 h period (Figure 4A). In the MVA pathway, acetyl-CoA C-acetyltransferase (ACAT) was slightly downregulated only at 3 h, while diphosphomevalonate decarboxylase (MVD) was apparently upregulated at 3 h and 6 h. Besides, isopentenyl-diphosphate delta-isomerase (idi) catalyzed the reversible conversion of IPP and DMAPP, and one of the idi isoforms in *N. laevis* was significantly upregulated at 3 h and 6 h. IPP and DMAPP are converted to form geranylgeranyl diphosphate (GGPP) catalyzed by the geranyl diphosphate synthase (GPPS), farnesyl diphosphate synthase (FDPS), and geranylgeranyl diphosphate synthase (GGPPS). Among these three enzymes, the gene encoding GGPPS had a relatively higher transcription level than the other two genes, and the gene encoding FDPS showed elevated expression under glucose addition. Therefore, the gene transcription levels of terpenoid backbone biosynthesis were overall upregulated by glucose addition.

In the carotenoid biosynthesis pathway, GGPP is condensed to the first 40-carbon carotene, phytoene, catalyzed by phytoene synthase (PSY). The phytoene is converted to lycopene after several desaturation and isomerization steps, mediated by phytoene desaturase (PDS), zeta-carotene isomerase (ZISO), and zeta-carotene desaturase (ZDS). The transcripts of PDS and ZISO was reduced upon glucose addition, and that of PSY and ZDS remained unchanged (Figure 4B). From the genetic engineering perspective, the reduction of PDS and ZISO correlated well the decrease of fucoxanthin content, thus the two enzymes might catalyze the rate-limiting steps in the pathway. Lycopene β-cyclase (lcyB) converts lycopene to β-carotene, and beta-carotene 3-hydroxylase (CHYB) catalyzes the hydroxylation of β-carotene to form zeaxanthin. In green algae, lycopene represents the branching point for α-carotene and β-carotene, and lycopene ε-cyclase (LCYe) diverts the carotenoid flux towards lutein instead of zeaxanthin [34]. However, in *N. laevis* the ortholog of LCYe is absent, which is consistent with previous studies suggesting LCYe gene is absent in diatoms, explaining why α-carotene and its derivatives are not found [35]. Zeaxanthin was epoxidized to violaxanthin by zeaxanthin epoxidase (ZEP) via the intermediate antheraxanthin, and the xanthophyll violaxanthin was reversely deepoxidized to zeaxanthin by violaxanthin de-epoxidase (VDE) (violaxanthin cycle). The latter conversion was essential for the photoprotection of plants or algae when exposed to excessive light [36]. In this study, three candidate ZEP isoforms and one VDE were identified, and one of ZEP isoforms (TRINITY_DN5319_c0_g1) was upregulated upon glucose addition.

Violaxanthin is the precursor of diadinoxanthin, diatoxanthin, and fucoxanthin, the major xanthophyll molecules in diatoms, and the diadinoxanthin–diatoxanthin cycle is an important short-term photoprotective mechanism [37]. However, the research on fucoxanthin biosynthesis in diatoms is still in its infancy, as the enzymes catalyze downstream conversions of violaxanthin has yet been identified in diatoms [38]. The structural studies on fucoxanthin and chlorophyll a/c binding proteins (FCPs) revealed that fucoxanthin-FCPs pigment-protein complex play both light-harvesting and photoprotection roles [23,24]. In *N. laevis*, 18 genes were annotated as putative genes encoding FCPs (Table S5), among which one gene was downregulated at 3 h, two genes were downregulated at 6 h and 12 h, while 1 gene was upregulated at 12 h.

### 2.5. Alterations to FA Metabolism during the Transition from Autotrophy to Mixotrophy

**Figure 5:**
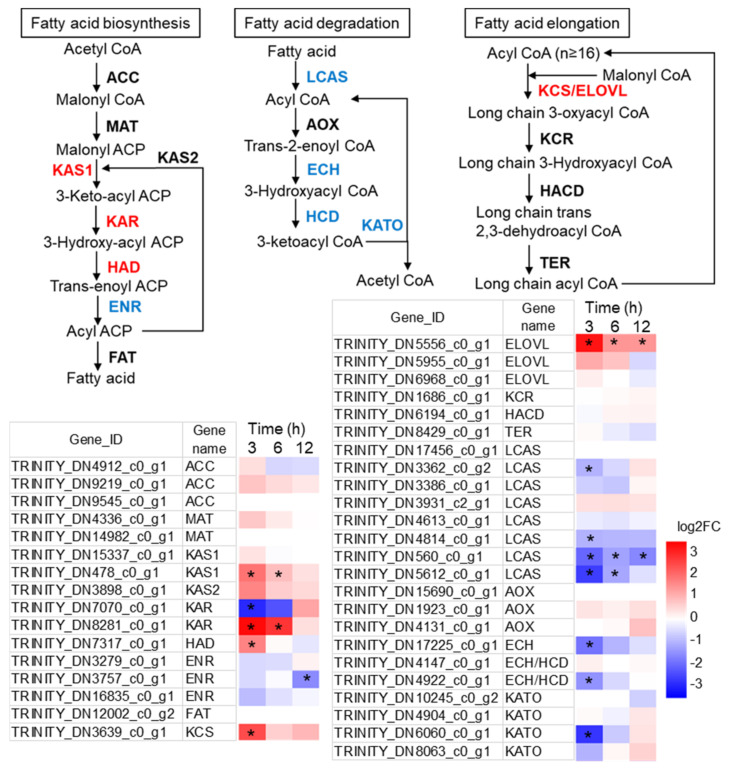
Expression patterns of genes involved in fatty acid metabolism. ACC, acetyl-CoA carboxylase; ACP, acyl carrier protein; MAT, malonyl-CoA:acyl carrier protein transacylase; KAS, 3-ketoacyl-ACP synthase; KAR, 3-ketoacyl-ACP reductase; HAD, 3-hydroxyacyl-ACP dehydratase; ENR, enoyl-ACP reductase; FAT, acyl-ACP thioesterase; LCAS, long-chain acyl-CoA synthetase; AOX, acyl-CoA oxidase; ECH, enoyl-CoA hydratase; HCD, 3-hydroxyacyl-CoA dehydrogenase; KATO, 3-ketoacyl-CoA thiolase; ELOVL, elongation of very long chain fatty acids protein; KCS, 3-ketoacyl-CoA synthase; KCR, very-long-chain 3-ketoacyl-CoA reductase; HACD, very-long-chain-3-hydroxyacyl-CoA dehydratase; TER, very-long-chain enoyl-CoA reductase. * indicates the expression level in +G group was significantly differential compared with control group (|log_2_FC| ≥ 1.00 and padj < 0.05). The red color of genes indicates upregulation and blue color indicates downregulation.

The first committing step of de novo fatty acid synthesis is the carboxylation of acetyl CoA to produce malonyl CoA catalyzed by acetyl-CoA carboxylase (ACC), which is a key enzyme in the de novo fatty acid synthesis [39,40]. The malonyl CoA is transferred to an acyl carrier protein (ACP) by the malonyl-CoA:acyl carrier protein transacylase (MAT) leading to the formation of malonyl-ACP for fatty acid synthesis. In this study, the transcription level of neither ACP or MAT genes had significant change between +G and −G group in *N. laevis* (Figure 5).

The subsequent condensation reactions from malonyl-ACP to C16 and/or C18 fatty acids involves a set of fatty acid synthases including 3-ketoacyl-ACP synthase (KAS, fabF and fabH), 3-ketoacyl-ACP reductase (KAR, fabG), 3-hydroxyacyl-ACP dehydratase (HAD, fabZ), and enoyl-ACP reductase (ENR, fabI, and fabK) [39]. In *N. laevis*, the transcription level of these enzymes (fabF, fabH, fabG2, fabZ) were overall upregulated in +G group at 3 h (Figure 5). Despite the fact that FabG1 was downregulated, its transcript abundance was much lower than FabG2, indicating the FabG2 rather than FabG1 was the primary functional isoform catalyzing the conversion of 3-Ketoacyl ACP to 3-Hydroxyacyl ACP.

The de novo synthesized fatty acids in the form of acyl-ACP can be released as free fatty acids by an acyl-ACP thioesterase (FAT), and ligated to CoA via a long-chain acyl-CoA synthetase (LCAS). The fatty acids in the form of acyl-CoA can be further elongated or oxidated. In microalgae, isoforms of LCAS played different roles involving in TAG biosynthesis or in fatty acid β-oxidation [41]. Among the eight putative LCAS genes in *N. laevis*, four genes were downregulated by glucose addition (Figure 5), in spite that their roles in lipid metabolism needed more detailed research in future. Fatty acids degradation involves a set of enzymes including LACS, acyl-CoA oxidase (AOX), enoyl-CoA hydratase (ECH), 3-hydroxyacyl-CoA dehydrogenase (HCD) and 3-ketoacyl-CoA thiolase (KATO). Overall, the expression of these genes involved in FA degradation were strongly repressed upon glucose addition, indicative of attenuated degradation of fatty acids under mixotrophic condition.

Fatty acids are de novo synthesized in the stroma, then converted into very long chain polyunsaturated fatty acids at the endoplasmic reticulum (ER). In FA elongation pathway, the transcription of two isoforms of Elongation of Very Long Chain Fatty Acid Proteins (ELOVL) were dramatically increased (TRINITY_DN5556_c0_g1 and TRINITY_DN5955_c0_g1), and 3-ketoacyl-CoA synthase (KCS) was 4.6-fold upregulated under mixotrophic condition. These two enzymes were known as responsible for extending palmitoyl-CoA and stearoyl-CoA to very-long-chain acyl CoAs. These two upregulated genes might be crucial for EPA biosynthesis; therefore, they are the potential targets for gene engineering to enhance EPA production.

### 2.6. Weighted Gene Co-Expression Network Analysis (WGCNA)

**Figure 6:**
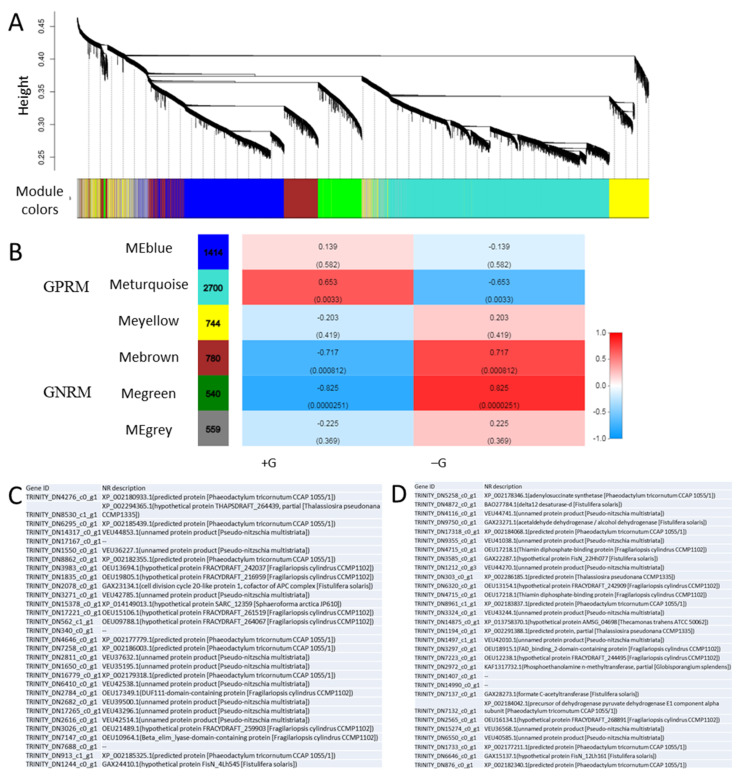
Weighted gene co-expression network analysis (WGCNA) in *N. laevis*. (**A**) Gene dendrogram and module colors based on gene transcript levels. (**B**) Correlation between modules and group traits (+G or −G). (**C**) Hub genes identified from glucose-associated positive regulatory module (GPRM). (**D**) Hub genes identified from glucose-associated negative regulatory module (GNRM).

To determine regulatory modules and the hub genes regulating the physiological responses related to glucose supplementation, we further carried out the WGCNA analysis using RNA-seq data of *N. laevis* (Figure 6). A total of 6 co-expressed gene modules were identified via hierarchical clustering based on gene transcript levels (Figure 6A). The correlation coefficients between group physiological traits and the identified gene modules were calculated to identify glucose regulatory modules (Figure 6B). The correlation efficient of the turquoise module and +G group is up to 0.65 with a *p*-value of 0.003, suggesting that the turquoise module was likely to be positively associated with glucose regulation. In contrary, the green module with a correlation efficient of −0.825 and a *p*-value of 2.51 × 10^−5^ was suggested likely to be negatively associated with glucose regulation. Therefore, the turquoise module and green module were choosing to carry out further analysis of hub genes, renamed as glucose-associated positive regulatory module (GPRM) and glucose-associated negative regulatory module (GNRM), respectively. The hierarchical cluster analysis of GPRM and GNRM showed that most genes in GPRM were strongly repressed upon glucose addition while most genes in GNRM were upregulated (Figure S4). Then, the hub genes analysis was performed, and 30 hub genes were identified from each module, respectively (Figure 6C,D). NR annotation showed that the function of most of these genes were unknown. In GPRM, there was one gene (TRINITY_DN2078_c0_g1) with high sequence homology to Cell Division Cycle 20-like protein 1 (CDC20) from Fistulifera solaris, and CDC20 is essential for the anaphase onset as a cofactor of the anaphase promoting complex/cyclosome (APC/C) controlling progression through mitosis and the G1 phase of the cell cycle [42,43]. Moreover, another gene (TRINITY_DN2784_c0_g1) from GPRM has the homology with a DUF111-domain-containing protein, which was likely associated to carbohydrate transport and metabolism [44]. In GNRM, several genes were identified as hub genes with putative function, including an adenylosuccinate synthetase (ADSS, TRINITY_DN5258_c0_g1), acetaldehyde dehydrogenase (ALDH, TRINITY_DN9750_c0_g1), a thiamin diphosphate-binding protein (ThDP-BP, TRINITY_DN4715_c0_g1), a formate C-acetyltransferase (TRINITY_DN7137_c0_g1), a pyruvate dehydrogenase E1 component alpha subunit (TRINITY_DN7132_c0_g1), a Phosphoethanolamine n-methyltransferase (TRINITY_DN2972_c0_g1) and a delta12 desaturase (D12D, TRINITY_DN4872_c0_g1). Among these genes, ADSS is an enzyme that plays an important role in purine biosynthesis and ALDH catalyzes the conversion of acetaldehyde to acetyl-CoA. Formate C-acetyltransferase catalyzes the reversible conversion of pyruvate and CoA into formate and acetyl-CoA, and is involved in the regulation of anaerobic glucose metabolism. The pyruvate dehydrogenase (PDH) complex is a mitochondrial multienzyme complex that catalyzes the overall conversion of pyruvate to acetyl-CoA and provides the primary link between glycolysis and the tricarboxylic acid (TCA) cycle. Phosphoethanolamine n-methyltransferase participates glycerophospholipid metabolism, and it is essential to phosphatidylcholine (PC) synthesis in Arabidopsis [45]. D12D is a key enzyme involved in the synthesis of omega-3 and omega-6 FAs, responsible for the conversion of oleic acid (OA, 18:1 n−9) to linoleic acid (LA) [46].

## 3. Materials and Methods

### 3.1. Algal Strain and Cultivation Conditions

The marine diatom *N. laevis* (UTEX 2047) was obtained from the Culture Collection of Algae at The University of Texas, USA. All cultures were grown in LDM medium in the 250-mL column (3-cm diameter) photoreactor with aeration of 1.5% CO_2_ enriched air. Continuous illumination at 40 μmol photons m^−2^ s^−1^ was applied. Cells were pre-cultivated photoautotrophically at 25 °C for 5 days. To induce trophic transition, cells from autotrophic cultures were inoculated into LDM medium with addition of 5 g/L glucose (+G) at the initial biomass concentration of ~0.4 g/L. Cultures under autotrophic condition were maintained as control (−G).

### 3.2. Lipids Analysis

For fatty acids (FAs) analysis, lyophilized algal cells were directly transesterificated with 1% (*v/v*) sulfuric acid in methanol and methylbenzene at 50 °C overnight, and analyzed using gas chromatography–mass spectrometry (GC–MS) equipped with a DB-WAX capillary column (30 m × 0.25 mm × 0.25 μm) (Agilent, Palo Alto, CA, USA). Helium was used as the carrier gas with the flow rate of 1.2 mL/min. Samples were injected in split mode (5:1 split ratio) at an oven temperature of 45 °C with an injection volume of 1 μL. The oven temperature was raised from to 45 °C 150 °C at 15 °C/min, then to 240 °C at 6 °C/min, and held at 240 °C for 8 min. Total lipids were quantified as the fatty acids contained in the total lipids, namely total fatty acids (TFA). EPA peaks were identified by the MS spectrum matching from database. Heptadecanoic acid (C17:0, Sigma-Aldrich, St. Louis, MO, USA) was used as the internal standard.

### 3.3. Polysaccharides Content Analysis

Microalgal cells were collected by centrifugation. Cells were homogenized by a tissue lyser at 30 Hz for 1 min. After homogenization, intracellular soluble polysaccharides were extracted in distilled water. Four times volume of ethanol was added for polysaccharides precipitation overnight. Then, the precipitates were collected and redissolved in distilled water for soluble polysaccharides analysis. The glucose was used as standard to draw a standard curve. One milliliter of 6% phenol and 5 mL of concentrated sulfuric acid were added to each tube. After mixing and reaction for 10 min at room temperature, samples were cooled to room temperature and measure the absorbance at 490 nm wavelength.

### 3.4. Pigments in Photosynthesis Complexes

Pigments were extracted from fresh algal cells using methanol. By measuring the absorbance, respectively, at 664 nm and 630 nm with a spectrophotometer (NanoDrop Technologies, Wilmington, Delaware, USA), chlorophyll concentration was calculated with the following equation [47]:

Chl *a* = 13.2654 × A664 − 2.6839 × A630 (1)

Chl *c* = 28.8191 × A630 − 6.0138 × A664 (2)

Identification and quantification of fucoxanthin were performed on ACQUITY Ultra Performance LC H-Class coupled in-line to a Xevo TQ-XS triple quadrupole mass spectrometer with a ESI probe (Waters, Milford, Massachusetts, USA). Samples were injected on a ACQUITY UPLC HSS T3 column (2.1 mm × 100 mm, 1.8 μm, Waters), and the eluents were acetonitrile (A), methanol (B) and demineralized water contained 0.1% (*v/v*) formic acid (C). The gradient was initiated from 58% A, 27% B, and 15% C, then transiently to 92%A and 8% C at 4 min, and finally reverted to its initial composition at 8 min followed by an equilibration phase of 2 min. The flow rate was at 350 μL/min. Nitrogen was used as desolvation gas (800 L/h at 400 °C). The spray capillary voltage was 3 kV for the positive ion mode. Multiple reaction monitoring (MRM) scanning mode was used for mass spectrometry detection. The standards of fucoxanthin were purchased from Sigma (St. Louis, MO, USA).

### 3.5. Transcriptomics Analysis

RNA sequencing (RNA-seq) experiment was performed on 18 samples collected at 3 h, 6 h, and 12 h after inoculation in treated medium with glucose addition (+G) and control group (−G), respectively. Total RNA was extracted using the plant RNA extraction kit (TaKaRa, Tokyo, Japan), and contaminating DNA was removed with RNase-free DNase I. The assessment of RNA concentration and quality was performed by Nano-Drop (ND1000 UV–Vis spectrophotometer; NanoDrop Technologies, Wilmington, Delaware, USA) and Agilent 2100 Bio analyzer (Agilent Technologies, Inc., Palo Alto, CA, USA). After purification, poly-A containing mRNA molecules were fragmented and reverse-transcribed into cDNA, then adaptor sequences were ligated to cDNA molecules. The products were purified and enriched with PCR amplification. The PCR yield was quantified by QuantiFluor^®^ dsDNA System, and samples were pooled together to form the final library. The pooled samples were subjected to cluster generation and sequenced using an Illumina HiSeq. Raw reads were trimmed and clipped with SeqPrep (https://github.com/jstjohn/SeqPrep, accessed on 20 January 2021) and Sickle (https://github.com/najoshi/sickle, accessed on 20 January 2021) software by filtering the low-quality reads, reads with adaptors and reads with unknown bases (N bases more than 5%) to get clean reads. The RNA-seq data were deposited in the Gene Expression Omnibus under accession number GSE168959.

De novo transcriptome assembly was performed with Trinity software (Version v2.8.5, https://github.com/trinityrnaseq/trinityrnaseq, accessed on 20 January 2021). The assembled transcriptome sequences were annotated using major databases (Non-Redundant Protein Sequence Database (NR), Swiss-Prot, Pfam protein family database, Clusters of Orthologous Groups (COG), Gene Ontology database (GO) and Kyoto Encyclopedia of Genes and Genomes (KEGG)), and statistics on its annotations were carried out in each database. RSEM software was used to calculate gene expression levels from RNA-seq data, and the expression profiles for all genes were normalized with the standardized transcript abundance measure “transcripts per million” (TPM) [48]. R studio (Version 4.0.2., Lucent Technologies Inc. (formerly AT&T Bell Laboratories), Murray Hill, NJ, USA) software was used to perform statistics: Pearson correlation between all samples was calculated using cor function, and PCA analysis was performed with all samples using princomp function. Finally, differential expressed genes (DEGs) were identified between samples and clustered with DEseq2 (Version 1.24.0, http://bioconductor.org/packages/stats/bioc/DESeq2/, accessed on 16 March 2021) software using the parameters as |log_2_FC| (Fold Change) ≥ 1.00 and adjusted *p* value (padj) < 0.05.

### 3.6. Weighted Gene Co-Expression Network (WGCNA)

For investigating correlations between genes and glucose-induced trophic transition, a global weighted gene co-expression network (WGCNA) were constructed as follows (https://horvath.genetics.ucla.edu/html/CoexpressionNetwork/Rpackages/WGCNA/, accessed on 16 March 2021): (1) genes and samples were first filtered by the variation efficient and hierarchical clustering analysis; (2) co-expressed gene modules were identified from the network using the function blockwise Modules; (3) modules with higher Pearson correlation coefficient were selected to perform the hub gene analysis.

### 3.7. Metabolomics Analysis

The cells were collected by centrifugation and quenched rapidly with liquid nitrogen. Cells were homogenized by a tissue lyser at 30 Hz for 1 min, and the adapter set of the tissue lyser was pre-chilled at −80 °C overnight before use. The metabolites were extracted by pre-chilled methanol: acetonitrile: H_2_O (2:1:1, *v/v/v*) after homogenized using a tissue lyser. The supernatant was collected and vacuum-dried using Centri Vap benchtop vacuum concentrator and re-suspended in methanol: H_2_O (1:9, *v/v*). Twenty microliters of each sample was taken and mixed into a quality control (QC) sample for evaluation of reproducibility and stability of the analysis.

Metabolomics analysis was performed with an UPLC (waters, USA) in-line with Q Exactive mass spectrometer (Thermo Fisher Scientific, Waltham, MA, USA) equipping with a BEH C18 column (1.7 μm, 2.1 mm × 100 mm, Waters, USA). The eluents were H_2_O containing 0.1% (*v/v*) formic acid (A) and methanol containing 0.1% (*v/v*) formic acid (B) for positive ion mode (ESI+), and H_2_O containing 10 mM ammonium formate (A) and 95% methanol containing 10 mM ammonium formate (B) for negative ion mode (ESI-). The linear gradient was as follow: 0–1 min, 98% A; 1–9 min, 98% A to 2% A; 9–12 min, 2% A; 12–12.1 min, 2% A to 98% A and maintained until 15 min. The flow rate was at 350 μL/min, and injection volume was 5 μL. MS data was recorded over the m/z range 70–1050. The flow rate of sheath gas was 40, and aux gas flow rate was 10. The spray voltage was 3.8 kV for positive ion mode and 3.2 kV for negative ion mode. The capillary temperature was 320 °C and the aux gas heater temperature was 350 °C. Data reprocessing was performed with Compound Discoverer 3.0 (Thermo Fisher Scientific, USA) and MetaX (http://metax.genomics.cn, accessed on 16 March 2021) [49].

### 3.8. Statistical Analysis

All the experiments were conducted in at least three biological replicates. Experimental results were expressed as mean value ± SD. The statistical significance of the results was tested by *t* test.

## 4. Conclusions

In conclusion, glucose addition affected the biochemical composition of *N. laevis*. The fucoxanthin and chlorophyll contents were significantly decreased, while EPA was increased. Transcriptomic and metabolomic analysis suggested that the impact of glucose-induced trophic transition include upregulation of enzymes associated with the pyruvate metabolism, amino acid biosynthesis, and fatty acid elongation. ELOVL and KCS are likely the main contributors to EPA accumulation, and the downregulation of PDS and ZISO is likely to be responsible for the decrease of fucoxanthin content. Moreover, hub genes were identified from two glucose regulatory gene module, GPRM and GNRM respectively, and their functions were associated with cell cycle, carbohydrate metabolism, purine biosynthesis, and lipid metabolism.

## Data Availability

Not applicable.

## Supplementary Materials

The following are available online at https://www.mdpi.com/article/10.3390/md19080426/s1, Figure S1: Transcriptome analysis overview during the transition from autotrophy to mixotrophy. (A) Principal component analysis (PCA) (B) The box plot of the gene expression level (C) Sequence similarity annotation in NR (Non-Redundant Protein Sequence) database. Figure S2: Hierarchical cluster and KEGG classification of most up-regulated and down-regulated differential expressed genes (DEGs) at 3 h. (A) hierarchical cluster analysis of the top 100 most up-regulated DEGs (B) Hierarchical cluster analysis of the top 100 most down-regulated DEGs (C) KEGG classification of the top 100 most up-regulated DEGs (D) KEGG classification of the top 100 most down-regulated. Figure S3: Metabolome analysis overview during the transition from autotrophy to mixotrophy. (A) The volcano plots of metabolites under positive ion mode (ESI+) (B) The volcano plots of metabolites under negative ion mode (ESI-) (C) KEGG classification of all metabolites. Figure S4: The hierarchical cluster analysis of glucose-associated positive regulatory module (GPRM) (A) and glucose-associated negative regulatory module (GNRM) (B). Table S1: The gene expression levels of all unigenes. Table S2: The Pearson correlation coefficients for all gene expression levels. Table S3: KEGG pathway classification of most up-regulated and down-regulated DEGs. Table S4: All peaks of Metabolomics identified and quantified. Table S5 Genes related in carotenoids biosynthesis and fatty acids metabolism.
