# Supplementary material for: Transcriptomics and Metabolomics Analyses Provide Novel Insights into Glucose-Induced Trophic Transition of the Marine Diatom Nitzschia laevis
Source: Mar Drugs. 2021 Jul 27;19(8):426. doi: 10.3390/md19080426 (PMC8401676; doi:10.3390/md19080426)
Supplement: Supplementary file 1 [file marinedrugs-19-00426-s001.zip › SupplementaryFigure.pdf]

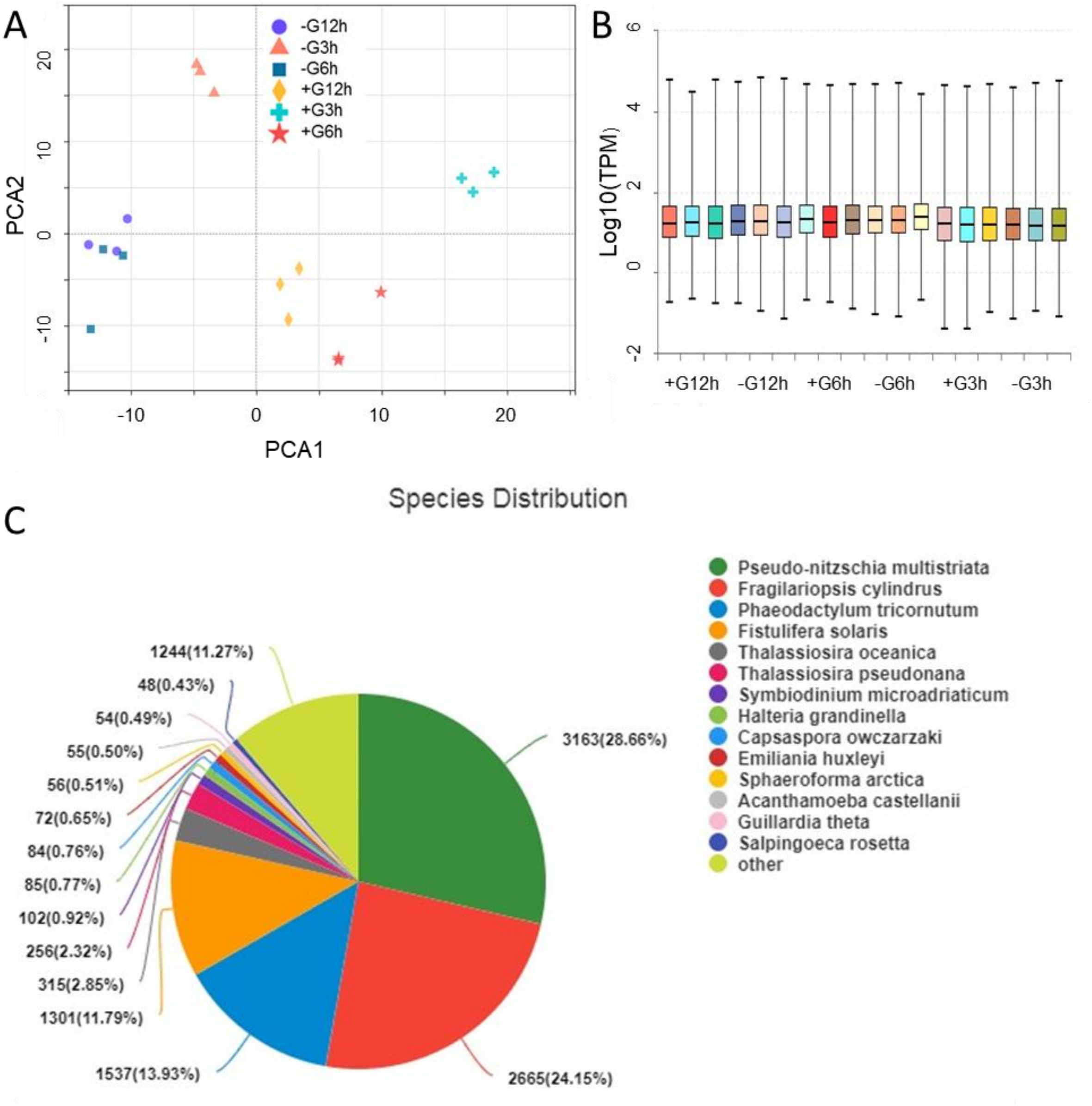

Figure S1 Transcriptome analysis overview during the transition from autotrophy to mixotrophy. (A) Principal component analysis (PCA) (B) The box plot of the gene expression level (C) Sequence similarity annotation in NR (Non-Redundant Protein Sequence) database.

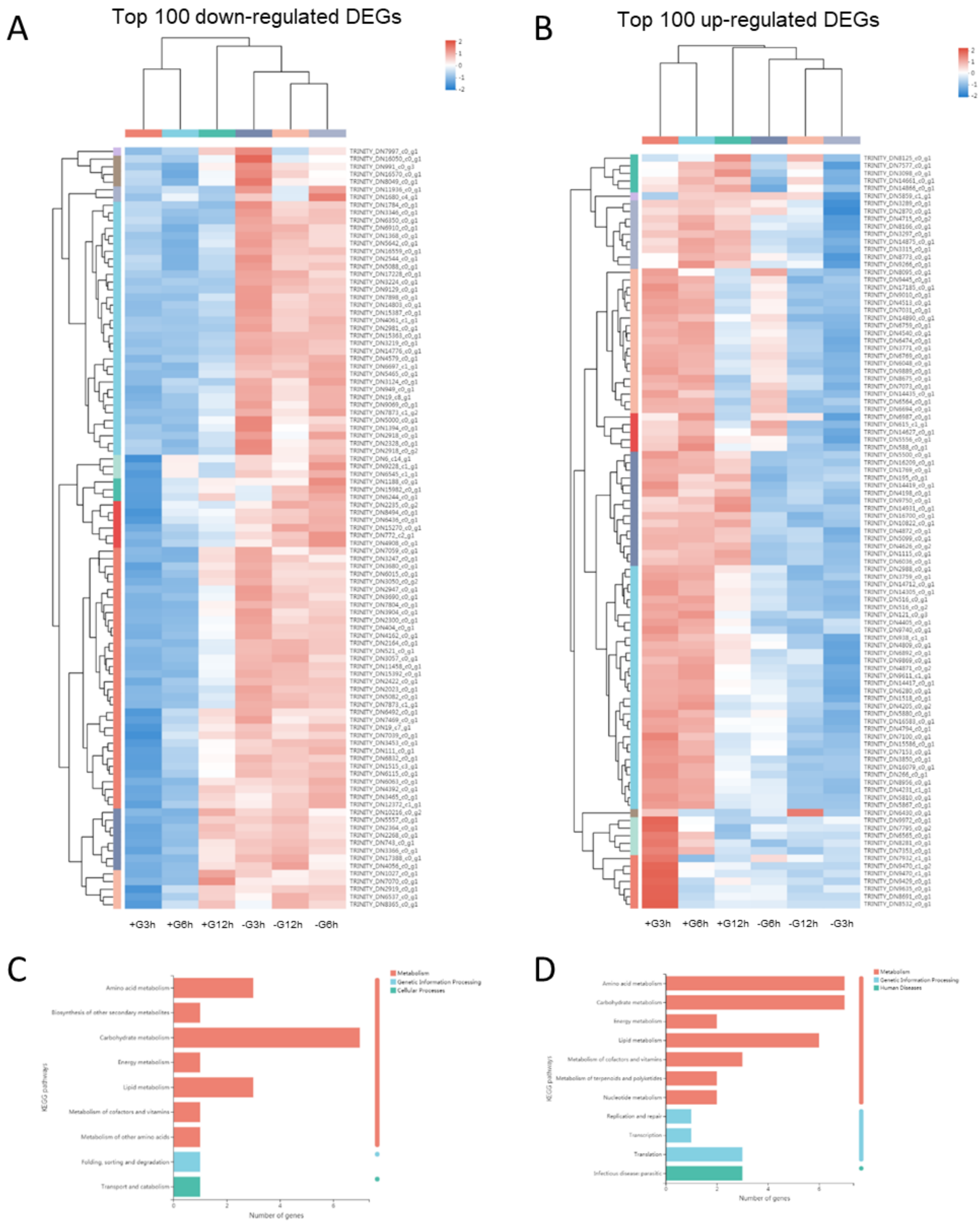

Figure S2 Hierarchical cluster and KEGG classification of most up-regulated and down-regulated differential expressed genes (DEGs) at 3 h. (A) hierarchical cluster analysis of the top 100 most up-regulated DEGs (B) Hierarchical cluster analysis of the top 100 most down-regulated DEGs (C) KEGG classification of the top 100 most up-regulated DEGs (D) KEGG classification of the top 100 most down-regulated.

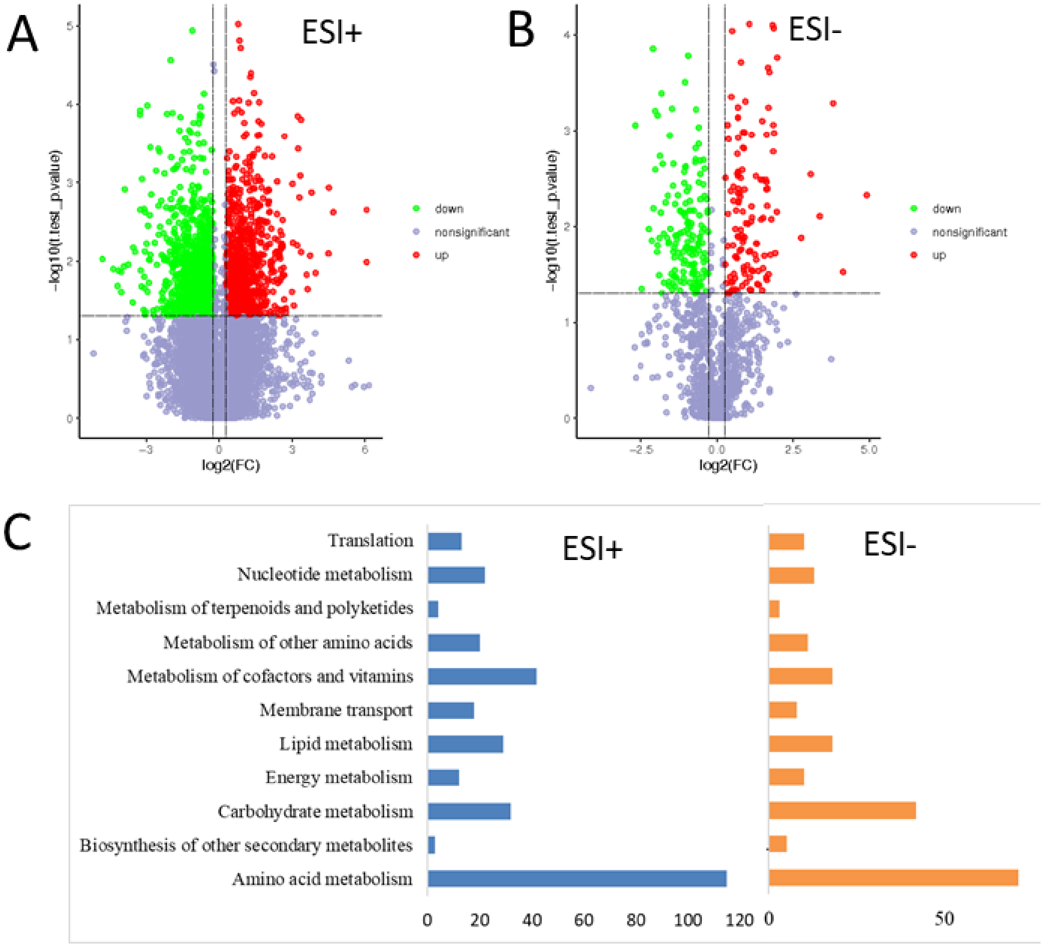

Figure S3 Metabolome analysis overview during the transition from autotrophy to mixotrophy. (A) The volcano plots of metabolites under positive ion mode (ESI+) (B) The volcano plots of metabolites under negative ion mode (ESI-) (C) KEGG classification of all metabolites.

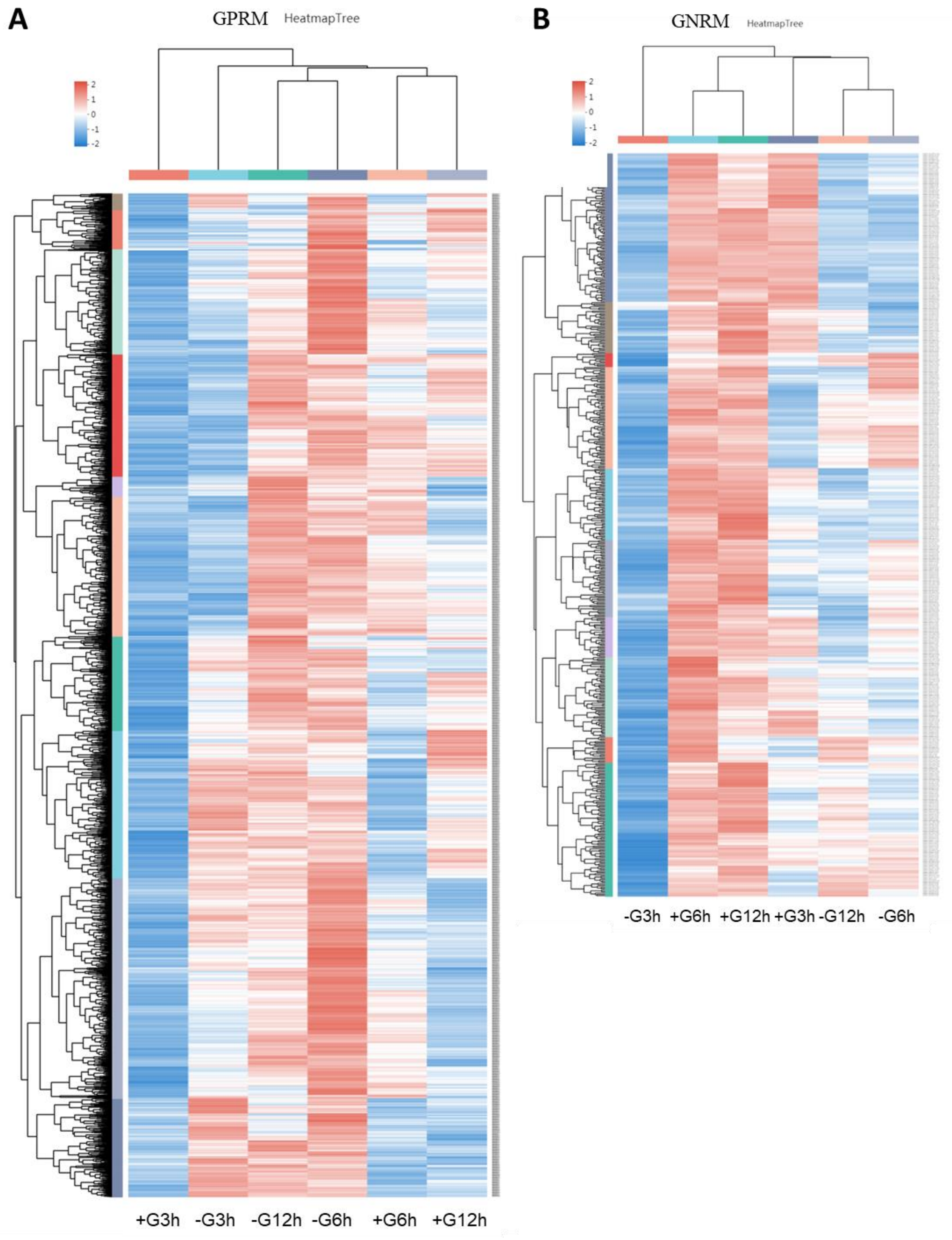

Figure S4 The hierarchical cluster analysis of glucose-associated positive regulatory module (GPRM) (A) and glucose-associated negative regulatory module (GNRM) (B).
